# Synaptic Plasticity Can Produce and Enhance Direction Selectivity

**DOI:** 10.1371/journal.pcbi.0040032

**Published:** 2008-02-15

**Authors:** Sean Carver, Eatai Roth, Noah J Cowan, Eric S Fortune

**Affiliations:** 1 Department of Psychological and Brain Sciences, Johns Hopkins University, Baltimore, Maryland, United States of America; 2 Department of Mechanical Engineering, Johns Hopkins University, Baltimore, Maryland, United States of America; Indiana University, United States of America

## Abstract

The discrimination of the direction of movement of sensory images is critical to the control of many animal behaviors. We propose a parsimonious model of motion processing that generates direction selective responses using short-term synaptic depression and can reproduce salient features of direction selectivity found in a population of neurons in the midbrain of the weakly electric fish Eigenmannia virescens. The model achieves direction selectivity with an elementary Reichardt motion detector: information from spatially separated receptive fields converges onto a neuron via dynamically different pathways. In the model, these differences arise from convergence of information through distinct synapses that either exhibit or do not exhibit short-term synaptic depression—short-term depression produces phase-advances relative to nondepressing synapses. Short-term depression is modeled using two state-variables, a fast process with a time constant on the order of tens to hundreds of milliseconds, and a slow process with a time constant on the order of seconds to tens of seconds. These processes correspond to naturally occurring time constants observed at synapses that exhibit short-term depression. Inclusion of the fast process is sufficient for the generation of temporal disparities that are necessary for direction selectivity in the elementary Reichardt circuit. The addition of the slow process can enhance direction selectivity over time for stimuli that are sustained for periods of seconds or more. Transient (i.e., short-duration) stimuli do not evoke the slow process and therefore do not elicit enhanced direction selectivity. The addition of a sustained global, synchronous oscillation in the gamma frequency range can, however, drive the slow process and enhance direction selectivity to transient stimuli. This enhancement effect does not, however, occur for all combinations of model parameters. The ratio of depressing and nondepressing synapses determines the effects of the addition of the global synchronous oscillation on direction selectivity. These ingredients, short-term depression, spatial convergence, and gamma-band oscillations, are ubiquitous in sensory systems and may be used in Reichardt-style circuits for the generation and enhancement of a variety of biologically relevant spatiotemporal computations.

## Author Summary

Short-term synaptic plasticity is ubiquitous in brain circuits, but its function in sensorimotor processing remains unclear. We propose a parsimonious model of motion processing using short-term depression to produce directionally selective responses. In the model circuit, information from two spatially separated receptive fields is combined after being asymmetrically processed by synapses that either exhibit short-term synaptic depression or do not. Motion in a preferred direction leads to a constructive interaction between the two channels; motion in the opposite direction does not. The model represents short-term synaptic depression as two processes with distinct time constants. The faster process alone suffices to generate direction selectivity in the circuit. The slow process, in contrast, can enhance direction selectivity to sustained stimuli. Therefore, the slow process mediates a form of attentional shift from alert, where the neuron responds more vigorously, to discriminating, where the neuron responds more selectively with fewer spikes. This explains a previously observed enhancement of direction selectivity in weakly electric fish in the presence of global synchronous gamma-band oscillations. These findings suggest a mechanistic connection between gamma-band oscillations and attention.

## Introduction

Control of animal behavior often requires the discrimination of the direction of movement of sensory images. In some behaviors, including tracking [1–5] and postural balance [6–9], animals must determine the direction of sensory slip to generate appropriate compensatory movements to stabilize the sensory image on the receptor array. For other behaviors, including prey capture [10,11], animals must respond to the direction of motion of prey relative to the sensory background. A neural correlate of these functions was described over 40 years ago: Hubel and Wiesel characterized central neurons in the mammalian visual system that exhibited preferential responses to particular directions of movement of sensory images [12–14]. Following this discovery, direction-selective response properties have been found in a diversity of animal species and across different sensory modalities, from the visual cortex of cats [12] and somatosensory cortex of monkeys [15] to the electrosensory midbrain of weakly electric fish [16,17].

Of particular interest are the direction selective responses of midbrain neurons observed in a species of weakly electric fish, Eigenmannia virescens. These neurons exhibit an unexpected enhancement of direction selectivity by the addition of concomitant naturally occurring sensory oscillations in the gamma frequency range [17]. These oscillations strongly induce short-term synaptic depression in these midbrain neurons, and measures of this depression correlate significantly with direction selectivity [17–19]. These correlations suggest that a depression-based mechanism might underlie both the generation of direction selectivity and its enhancement by the addition of gamma-band oscillations. Here we propose a parsimonious model to describe and explore the essential features of this mechanism.

**Figure 1:**
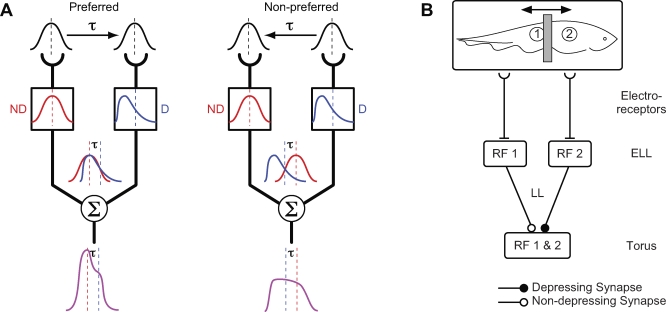
Short-Term Synaptic Depression Can Contribute to Direction Selectivity in an Elementary Reichardt Detector (A) Information from two channels (blue and red: right and left, respectively) converges on a post-synaptic cell. The blue channel exhibits short-term synaptic depression (D), which nonlinearly filters the stimulus, advancing the timing of the channel's peak response relative to the timing of the peak of the stimulus (blue dashed lines). The red channel exhibits no depression (ND), which linearly scales the stimulus, leaving the timing of the channel's peak response to coincide with the timing of the stimulus (red dashed lines). Differences in temporal processing interact with a spatial separation of the two channels' receptive fields to produce direction selectivity. A moving stimulus pulse first activating the red channel leads to the summation of coincident peaks in the post-synaptic cell. A stimulus pulse moving in the opposite direction leads to the summation of disparate peaks and hence a weaker response in the post-synaptic cell. (B) A schematic outline depicting the ascending electrosensory system in *Apteronotus* and *Eigenmannia* and other Gymnotiform genera. In short, electrosensory information from receptors in the skin (RF 1 and RF 2) project topographically onto the electrosensory lateral line lobe (ELL). Neurons in the ELL in turn project topographically onto neurons in the torus semicircularis (Torus) in the midbrain via the lateral lemniscus (LL). Midbrain afferents include both depressing and nondepressing synapses that converge on to individual neurons: this convergence of information meets the requirements for the proposed elementary Reichardt motion detector. We used three categories of stimuli: global stimuli (social signals that stimulate the entire sensory surface simultaneously), a localized moving bar (shown), and a larger moving sinewave grating.

The model is based on a conceptual framework for motion processing known as Reichardt detectors: information from two spatially separated channels with asymmetric temporal properties combine via a nonlinear operation on a downstream (post-synaptic) neuron to produce direction selective responses [20,21] (Figure 1). When supplied with a stimulus moving in the preferred direction, the temporal shift can compensate for the spatial separation, allowing the inputs from the two channels to interact constructively. The temporal phase shift is typically modeled as a pure delay or a low-pass filter.

For direction-selective neurons in V1, Chance et al. [22] propose that the dynamical differences between synapses that exhibit short-term synaptic depression and those that do not may provide a mechanism for generating both the asymmetrical temporal properties and the nonlinear operation required by an elementary Reichardt circuit (the electrosensory midbrain of *Eigenmannia* also exhibit these requisite ingredients for Reichardt-style selectivity based on short-term depression [19,23].) For a depressing synapse, the magnitude of the response in the post-synaptic cell decreases during repetitive activation [24–27]. Short-term synaptic depression involves two dynamic processes with distinct time constants: the faster process, with a time-constant on the order of tens to hundreds of milliseconds, can be attributed to the depletion of the supply of readily releasable synaptic vesicles, while the slower process, with a time-constant of seconds to tens of seconds, can be attributed to the mechanisms for the replenishment of this supply [25,26].

Here we propose a parsimonious model that describes a mechanistic linkage between short-term synaptic depression and direction selectivity, based on a Reichardt-style circuit. We further test the possibility that the enhancement of direction selectivity by concomitant gamma-band oscillations may be mediated by short-term synaptic depression. Global synchronous oscillations in activity may arise endogenously, as occurs in cortical and other circuits [28], or exogenously, as occurs in weakly electric fish from the interaction of the electric fields of nearby conspecifics [29] and the jamming avoidance response [30]. In the model, these oscillations induce depression, which can lead to an enhancement of direction selectivity to moving objects. We systematically explore the effects of variations of biologically relevant parameters of the model and evaluate the results in relation to electrophysiological data from a population of motion-sensitive electrosensory neurons in the midbrain of weakly electric fish [17].

## Results

Information from two spatially separated receptive fields converges onto a post-synaptic neuron via dynamically different synapses: one that exhibits short-term synaptic depression and the other that does not (Figure 1). The spatial separation of the receptive fields combined with the differences in temporal dynamics of the synaptic inputs satisfies the requirements for an elementary Reichardt motion detector. In this model, only the depressing synapse contributes state to the model, which consists of one or two variables whose dynamic evolution is governed by uncoupled and identical (up to parameters) nonlinear ordinary differential equations.

### Direction Selectivity Is Mediated by the Fast Process

**Figure 2:**
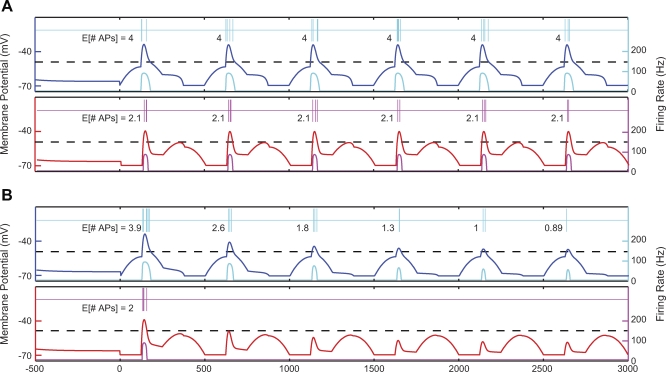
Response of Model to a Moving Sine Grating Following 500 msec Initialization with 50% Grey (A) The one-state model with the fast process alone, *s* = 1. (B) The two-state model with both the fast and slow processes, *s* = 0.99. Blue and red (middle trace of each subpanel) shows membrane potential, *V*_∞_(*t*). Cyan and magenta shows firing rate, *F*(*t*) (beneath *V*_∞_(*t*), and timing of action potentials determined from *F*(*t*) by a Poisson process (above). Blue and cyan (top subpanel in both A and B): preferred direction (σ = 1); red and magenta (bottom subpanel): non-preferred direction (σ = −1). Small numbers are the expected action potentials in each cycle. Black dotted line indicates threshold for action potentials. In the one-state model, the direction index (defined in Model) is constant across all cycles at 0.48. In the two-state model, the direction index increases from 0.49 to 1—the two-state model becomes more direction selective with time. Other parameters of model are *R_b_* = 5 Hz, *R_c_* = 172 Hz × log(67 × 0.2), *x_n_* = −45°, *x_d_* = 45°, *t_D_* = 150 msec, *t_S_* = 3,000 msec, *d* = 0.4, *g_d_* = 15, *g_n_* = 0.7,*V*_0_ = 0.7, *V*_0_ = 70 mV, *V_E_* = 0 mV, *V_t_* = −64 mV, *V_r_* = −65 mV, t*_m_* = 30 msec, t*_R_* = 10 msec. Other parameters of the stimulus are *t*_0_ = −500 msec, *t_s_* = 0 msec, *t_f_* = 3,000 msec, *f* = 2 Hz, *p*_0_ = ∞, *p*_1_ = −∞.

We have found that the one-state model, which includes only the fast process of short-term synaptic depression, exhibits direction selectivity (Figure 2A). Since short-term synaptic depression creates a phase advance in the synapse, a moving stimulus that first passes through the nondepressing area leads to a simultaneous arrival (a constructive combination) of signals from both synapses (Figure 2A, blue). Movement in the opposite direction leads to asynchronous arrival of information (Figure 2A, red). These results are similar to those reported previously [22].

The response to the sine-wave grating is nearly identical from cycle to cycle over time. In this case, the time constant of depression is fast relative to the period of the stimulation so that the depressing synapse has sufficient time to return to its initial state during the dark phase of each cycle.

### Enhancement Is Mediated by the Slow Process

In the two-state model, which includes both the fast and slow processes associated with short-term synaptic depression, neurons exhibit direction selectivity that enhances from cycle to cycle of a sustained sine wave grating (Figure 2B). In the first cycle, the response is nearly identical to the response of the one-state model. However, in each subsequent cycle there is a total reduction in the probability of firing and in the total number of spikes for both directions of motion. This reduction in probability of firing is asymptotic.

This overall reduction in firing nonetheless increases the direction index (reported in the caption to Figure 2 and defined in Model) by increasing the relative difference between the responses to the preferred and non-preferred directions of movement. Intracellularly, this enhancement effect will occur as long as the stimulus is maintained even if the depression limits the PSPs so that they do not reach the spiking threshold. In extracellular recordings, however, there is a possibility that the depression could lead to a complete elimination of spiking responses to the moving stimulus.

### Responses to Intermittent Stimuli

The sine-wave gratings that we used are sustained stimuli—such stimuli that might arise during image-stabilization tasks. In contrast, many behaviors, such as prey capture, involve spatiotemporally localized, or intermittent stimuli. We have examined the performance of the model to this class of stimuli.

**Figure 3:**
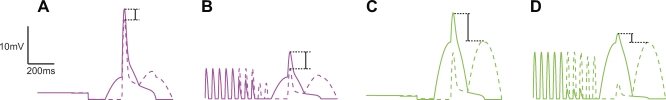
Response, *V*_∞_(*t*), of the Two State Model to a Pulse Stimulus (A,C) Initialized with a temporally and spatially uniform 50% grey stimulus. (B,D) Initialized with global synchronous gamma-band oscillations. Solid lines represent responses to movement in the preferred direction, dashed lines to the non-preferred direction. The responses in each plot are aligned to the peak of the PSP from the depressing synapse. This facilitates the comparison between the preferred and non-preferred directions of movement, but as a result, the onsets of the moving stimuli are not aligned. In the preferred direction, the object passes first through the nondepressing part of the receptive field and then enters the depressing part. Parameters of the model for this and all remaining figures are the same as listed in Figure 2 except for the synaptic factors, which vary. For A and B (magenta): g*_n_* = 0.2, g*_d_* = 8. For C and D (green): g*_n_* = 0.6, g*_d_* = 4. The stimulus parameters used throughout for the pulse stimulus are *f_g_* = 20 Hz, *A*_g_ = 0.7, or *A*_g_ = 0 (respectively, with or without initializing global synchronous oscillations), *t*_0_ = −1,000 msec, *t_s_* = 0 msec, *t_f_* = 5,500 msec, *ϕ* = 135°; σ, *p*_0_, and *p*_1_ differed depending on direction of movement: σ = 1, *p*_0_ = −16p, *p*_1_ = −19p, (preferred direction); σ = −1, *p*_0_ = −15p, *p*_1_ = −19p (non-preferred direction).

Our intermittent stimulus consists of the temporal sequence defined by Equation 2, which is a 1.5 cycle sine-wave pulse. Prior to the arrival of the stimulus, we initialized the system with at least 3 seconds of a spatially homogeneous stimulus of intermediate intensity, which we call 50% grey (see Model). At the arrival of the pulse, the model lies in approximately the same state as it does for the first cycle of the sine grating. As a result, the responses to the first cycle of the grating and to the intermittent stimulus are nearly identical (compare Figures 2B and 3A). For the same parameter values, the responses differ only because the stimuli are subtly different: the sine grating stimulus appears in both receptive fields at the same time, but at different phases, whereas the 1.5 cycle pulse first appears in one receptive field then moves to the other and disappears.

We also tested the model's response to an intermittent stimulus that was initialized not with a uniform background but rather with global synchronous gamma-band oscillations. These sorts of oscillations occur exogenously in groups of weakly electric fish [29] and endogenously in many CNS circuits [28]. In the model, the gamma-band oscillations drive activity simultaneously in both afferents which activates both the fast (0 < *D*(*t*) ≪ 1) and slow (0 < *S*(*t*) ≪ 1) processes in the depressing synapse (see Model).

The response of the model to the moving pulse after 3 seconds of global stimulation compares to its asymptotic response to a persistent sine grating (compare Figures 2B and 3B). The response in this condition is more “sparse” than in the grey-initialized condition—the responses are reduced due to the activation of the slow process associated with short-term synaptic depression. The code is more sparse in that fewer spikes more reliably encode information—the direction of movement. Depending on the values of the parameters, this reduction in spiking can lead to an enhancement of direction selectivity (Figure 3A versus 3B) or a reduction of the direction selectivity (Figure 3C versus 3D).

**Figure 4:**
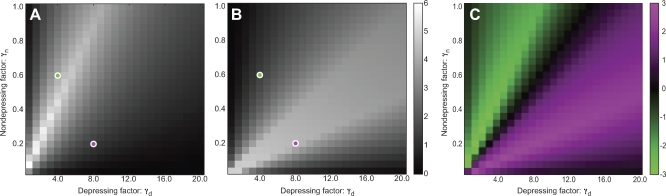
Magnitude of Direction Selectivity to a Moving Pulse Stimulus as a Function of Synaptic Factors (A) Initialized with 50% grey. (B) Initialized with Global Synchronous Gamma-Band Oscillations. Direction selectivity is quantified, in each condition, as the ratio (in decibels) of the peak values of *V*_∞_(*t*) – *V*_0_ in response to the pulse in the preferred to the non-preferred directions (solid and dotted curves in Figure 3). Brighter shades of grey indicate more direction selectivity. Colored circles indicate parameter values used in Figure 3. (C) Magnitude of enhancement (magenta) or reduction (green) of the directional response caused by the addition of global synchronous gamma-band oscillations. This measurement was quantified by the difference between the values calculated for (A) and (B). The color indicates sign of difference; intensity indicates magnitude.

We varied the contributions of the depressing and nondepressing synapses in the model and measured the response to the moving pulse in both the grey initialized and gamma-band initialized conditions (Figure 4A and 4B). Both plots show that direction selectivity reaches a maximum along a ray from the origin corresponding to an optimal ratio of depressing to nondepressing synapses.

To determine under which conditions the gamma-band initialization will lead to an enhancement of direction selectivity, we subtracted the surfaces in Figure 4A and 4B. The maximum enhancement was found to occur along a ray in which the depressing synapses make a greater contribution than the nondepressing synapses (Figure 4C, magenta). In addition, we found a region in which the combinations of depressing and nondepressing synapses lead to a reduction in direction selectivity with the addition of the gamma-band oscillations (Figure 4C, green).

### Relations between Measures of Direction Selectivity and Post-Synaptic Potential Depression

**Figure 5:**
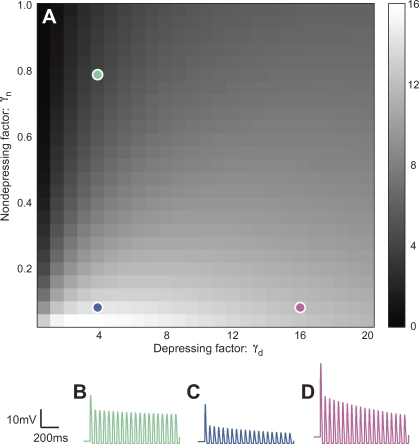
PSD Depression as a Function of Synaptic Factors and Exemplary Traces Used To Calculate This Measure (A) Measure of PSP depression (measured at the soma—a sum of all synaptic inputs) as a function of synaptic factors. Brighter shades of grey indicate more synaptic depression. (B–D) Exemplary traces of *V*_∞_(*t*) in response to the initializing global synchronous oscillations at three positions in parameter space (indicated in (A) with dots of corresponding colors). This measure of PSP depression is identical to that used in previous studies of midbrain neurons in *Eigenmannia* [17–19]. Specifically, depression was quantified as the ratio (in decibels) of the peak value of *V*_∞_(*t*) − *V*_0_ in the first cycle of the initializing global synchronous stimulation to the average of the peak values for 11 consecutive cycles, starting 3 seconds into the stimulus.

In intracellular recordings of midbrain neurons in *Eigenmannia*, the addition of an exogenous gamma-band oscillation resulted in an enhancement of direction selectivity to a moving bar stimulus [17]. In many neurons a correlate of the activity of inputs that experience short-term synaptic depression was observed: the amplitude of post-synaptic potentials (PSPs) declined on a cycle-by-cycle basis to a sustained gamma-band oscillation [17] (Figure 5). This “PSP depression” has been shown likely to result from short-term synaptic depression and not other mechanisms [18,19]. We tested the model with identical stimuli and made an identical measurement of “PSP depression” [18]. PSP depression is the magnitude of the decline in amplitude of PSPs measured at or near the soma, and is therefore a sum of the synaptic activity, including both depressing and nondepressing synaptic inputs, to the neuron (Figure 5).

In the model, PSP depression was strongest where the ratio of depressing synapses to nondepressing synapses was high, but the total number of synapses was low (Figure 5). Surprisingly, adding more depressing synapses actually decreased the measure of short-term synaptic depression. This measure consisted of a ratio of the response (maximum depolarization above resting potential) to the first cycle of global synchronous stimulation to the average responses to later cycles, after the transient had decayed. As more depressing synapses were added, both the numerator and the denominator of this ratio increased, but they did so in a way such that the value of this ratio decreased.

**Figure 6:**
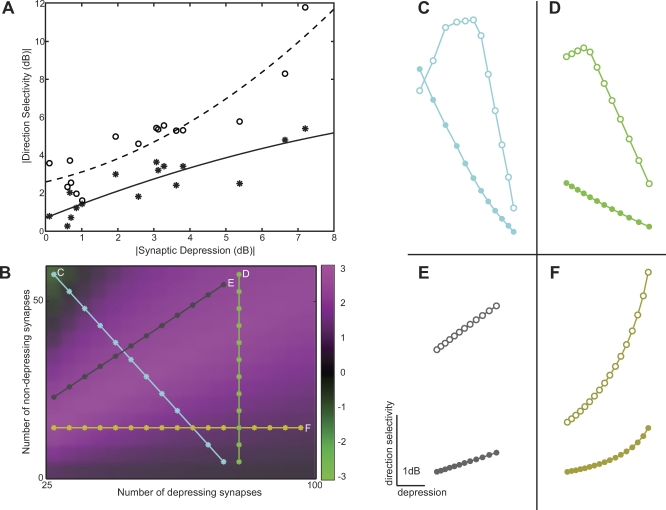
Reproducing the Properties of a Population of Midbrain Neurons (A) Experimental data from a population of midbrain neurons in *Eigenmannia* (replotted from [17]). PSP depression plotted against direction selectivity observed in two conditions for each neuron. Asterisks with solid line: response of each neuron to the moving object. Circles with dashed line: response of the same neurons to the moving object but with concomitantly presented global synchronous gamma-band oscillations. Curves are the best fit second degree polynomials. (B) Hypothetical distributions of synaptic properties in model populations of neurons. The background gradient is a region of Figure 4C where the addition of global synchronous oscillations enhances direction selectivity. Lines represent one-dimensional restrictions of the parameter space—these lines are four hypothetical distributions of parameter values across a population of neurons, lines labeled C through F. (C–F) Model data plotted in the same manner as the experimental data shown in (A). The data shown in each of the plots in (C–F) correspond to the labeled lines in (B). In (C–F), the lower curve shows direction selectivity to the moving object and the upper curve shows direction selectivity to the moving object with concomitant oscillations. For this plot, the parameters determining the relative contributions of the depressing and the nondepressing synapses have been expressed as a count of more numerous, but individually weaker, synapses. We arbitrarily model the numbers of depressing and nondepressing synapses, as well as their contributions to the post-synaptic potentials as roughly equal. To achieve these approximate equalities, we multiplied the number of the depressing synapses by a weight of 0.2 and the nondepressing synapses by a weight of 0.01. The hypothetical distribution are: (C) constant total number of synapses (80) with variable ratio of numbers of depressing to nondepressing; (D) constant number of depressing synapses (80) with variable number of nondepressing synapses; (E) constant ratio of numbers of depressing to nondepressing synapses (5/3) with variable total numbers of synapses; (F) constant number of nondepressing synapses (12) with variable number of depressing synapses. (E) and (F) are most similar to the experimental data shown in (A).

In *Eigenmannia*, strong correlations were observed between the magnitude of PSP depression measured in each neuron and the magnitude of direction selectivity to the moving bar in both grey and gamma-band initialized conditions (Figure 6A). We tested whether any simple set of parameters in the model could reproduce these relations.

We considered four hypothetical distributions, asking if each reproduced the qualitative observations drawn from the sample of neurons within the electrosensory midbrain of Eigenmannia virescens [17]. The qualitative observations were that the measures of PSP depression and direction selectivity were positively correlated in both grey and gamma-band initialized conditions, and that direction selectivity was increased by the addition of the gamma-band oscillation (Figure 6A).

We tested hypothesized distributions supported on one-dimensional restrictions of the parameter space in which 14 of the 16 model parameters remained fixed and the other two varied. The two parameters we varied determined the contributions of the depressing and nondepressing synapses. For convenience, we describe our parameter space restrictions in terms of numbers of synapses (with fixed synaptic weights, see Model). Distribution 1 assumes that the total number of synapses remains constant (80), but the ratio of depressing to nondepressing synapses varies. Distribution 2 assumes the number of depressing synapses remains constant (80) but the number of nondepressing synapses varies. Distribution 3 assumes the ratio of depressing to nondepressing synapses remains constant (5/3) but the total number varies. Finally, distribution 4 assumes the number of nondepressing synapses remains constant (12) but the number of depressing synapses varies. The restrictions of parameter space associated with these distributions are plotted in Figure 6B.

The resulting relationships between the measures of PSP depression and direction selectivity for each distribution are plotted in Figure 6C–6F. Distributions 6E and 6F match the three qualitative features seen in the population of neurons observed in the midbrain of *Eigenmannia*.

## Discussion

We examined the roles of short-term synaptic depression in the generation and enhancement of direction-selective responses. A one-state model that includes the fast process (hundreds of milliseconds) associated with short-term synaptic depression can produce direction selectivity in an elementary Reichardt motion detector. The addition of a second state, the slow process of short-term depression (seconds to tens of seconds), can lead to an enhancement of direction selectivity. This enhancement is a form of sparsification: fewer spikes more accurately encode direction of movement.

### Modulation of Spatiotemporal Processing via the Slow Process

In our model, the activation of the slower process of short-term depression is quantified by the state variable *S*(*t*) that depends upon the stimulus history. If there has been little recent stimulation (recent with respect to the time constant, which in this model is set to 3 seconds), then the neuron resides in a state in which it responds vigorously to stimuli that are moving in any direction.

On the other hand, if there has been recent stimulation, the depressing synapses will be depressed, and as a result the neuron will respond less vigorously. As shown in Figure 4C, the ratio of the contributions of depressing and non-depressing synapses determines whether the neuron will be more or less directionally selective in the depressed state that results from recent stimulation. In a population of midbrain neurons in *Eigenmannia*, recent stimulation leads to an enhancement of direction selectivity [17].

Recent stimulation shifts depressing synapses from a highly responsive state to a more depressed state. The difference between these two states may correspond to vigilance and focus (at least in *Eigenmannia*) and can be seen as a form of attention. In this way, the current value of the slow variable *S*(*t*) corresponds to an attentional state associated with the neuron. Indeed, the state of the slow process could be critical to specific computations. Although we only explored the responses to moving stimuli, these results could be applied more broadly to any computation in the brain that involves the temporal comparison of information that converges from independent pathways.

Indeed, we have shown that any stimulus that activates the slow process can lead to a shift in the computational properties of elementary Reichardt circuits. As a result, any change in the activity patterns, whether they be stimulus-driven or endogenous, could affect neural computations through similar mechanisms. This feature may provide an opportunity for animals to use behavior to modulate computations in the brain.

### Behavioral Modulation of Direction Selectivity

Animals may modulate the state of their synapses and hence the degree of direction selectivity in central neurons using behavior. This form of behavioral modulation requires that: 1) the behavior generates patterns of activity that elicit short-term depression in downstream neurons and 2) that these patterns of activity do not interfere with the motion processing.

Evidence for the behavioral modulation of direction-selectivity is seen in the Jamming Avoidance Response (JAR) of weakly electric fish. In the JAR, the electric fields of fish that are within about a meter of each other interact to produce oscillations in electroreceptor activity across the entire body [30]. In this way, the stimulation leads to global, synchronous activity across the receptor array. In the wild, the frequencies of these oscillations are most commonly in the gamma frequency band, from 20 to 80 Hz [29]. Laboratory experiments have shown that lower frequency oscillations, below approximately 8 Hz, impair the perception of moving objects [31–33].

These gamma oscillations are encoded by electroreceptors and propagate through the ascending electrosensory system. In the midbrain, these oscillations match the stimulation frequencies that best elicit short-term depression [18,19]. As a result, the ongoing oscillations that occur in social situations dramatically modulate short-term depression, leading to an enhancement of direction selectivity to intermittent stimuli [17].

Another more general example of behavioral modulation of temporal processing in plasticity-based elementary Reichardt circuits could include movement-induced self-stimulation of sensory receptors. For example, were an electric fish to remain motionless in a tube, the slow process will be in its initial state for midbrain electrosensory neurons, whereas if the fish were to move back and forth within the tube, the slow process may be activated. The neurons would be more responsive and less selective while the animal remained motionless and would be less responsive but more selective when the animal was moving relative to nearby objects.

### Intrinsic Modulation of Spatiotemporal Computations

If behavior can be used to generate patterns of brain activity that change the state of depressing synapses to alter spatiotemporal computations in the brain, then intrinsic activity in brain circuits could possibly have the same effect. The intrinsic activity would have to have two properties: 1) the patterns of activity must elicit short-term depression in downstream neurons and 2) these patterns of activity must not interfere with the spatiotemporal computation.

There are numerous examples of endogenous oscillations that occur at all levels of the CNS [28]. If the output of these endogenous synchronous oscillations converge on neurons that perform spatiotemporal computations, such as motion processing, through synapses that experience short-term synaptic depression, then the endogenous oscillations could have the same effects on computation that have been reported in *Eigenmannia*. The correlation between attentional processes and the emergence of gamma-band oscillations in cortical and other circuits may support this idea. Perhaps the gamma-band synchronous activity shifts elementary Reichardt circuits from a more responsive but less selective state to a less responsive but more selective state.

### Parameter Distributions in Populations of Neurons

The population of neurons observed in the midbrain of *Eigenmannia* showed a positive correlation between a measure of short-term synaptic depression (PSP depression) and direction selectivity. We tested the hypothesis that this relation follows from the proposed elementary Reichardt circuit that uses short-term synaptic depression. The model, however, clearly demonstrated that this relation is but one possible outcome. Indeed, without assumptions constraining the distribution of parameter values, the model does not make any specific prediction about the relationship between PSP depression and direction selectivity in populations of neurons.

Thus, the relationship previously observed in the midbrain of weakly electric fish [17] is likely associated with functional constraints beyond the elementary Reichardt circuit. These functional constraints may be related to the control of specific behaviors. For example, in tracking behavior [4,5,34], fish make compensatory movements to stabilize an image on the sensory array. Future studies will determine, via neural system identification, how a population of direction-selective neurons may encode the sensorimotor transfer function inferred from behavioral performance [5].

### Dynamic Receptive Field Structure

A key feature of the model is the convergence of information from spatially separated locations on the receptor array. The receptive field of a neuron that uses the mechanisms associated with this model should be composed of regions that differ in relation to short-term synaptic plasticity: the response of the neuron to stationary, highly localized stimuli at different locations should show differences in measures of short-term depression.

In the simplest case, the receptive field has two regions, e.g., caudal and rostral. If the neuron exhibits little or no short-term depression when the stimulus is in the caudal region and strong depression to local stimulation in the rostral region, then the model predicts that the neuron will respond more strongly to caudal-to-rostral movement than to rostral-to-caudal movement [23]. Preliminary evidence obtained from midbrain neurons in weakly electric fish are consistent with this hypothesis: gamma-band stimulation in subregions of the receptive fields of midbrain neurons elicit different levels of short-term synaptic depression (personal observations). Nevertheless, one could envision far more complicated receptive field structure leading to direction selectivity using similar mechanisms.

### Quantitative Validation of the Model

By posing a parsimonious model describing the transformation of the spatiotemporal stimulus into the neural response, we have taken the first step in a rigorous identification of the underlying neural system. The steps remaining include (for each neuron) validating or falsifying the model, estimating its parameters and comparing our model to alternative models. The field of system identification offers systematic and rigorous approaches to these remaining problems. For example, the stochastic model that includes action potential timing can be used to determine the likelihood that an experimentally observed train of action potentials was generated using mechanisms captured by the model. Systematic exploration of the parameters can be made to achieve the maximum likelihood estimates [35,36]. This procedure can be repeated for each neuron studied in the population to get more rigorous estimates of the distribution of parameter values.

## Model

The structure of the model is generic: it does not incorporate any specific features from particular animal systems. Nevertheless, the basic structure of the model is inspired by a model for direction selectivity in V1 neurons by Chance et al. [22]. Further, stimuli (including moving objects and global synchronous gamma-band oscillations) approximate those used in electrophysiological experiments in weakly electric fishes, such as Apteronotus leptorhynchus and Eigenmannia virescens [37]. The parameters of the model, including the time constants for the fast and slow processes of depression, are similar to those observed in mid-brain neurons in *Eigenmannia* [18].

Electrosensory information from receptors in the skin project topographically onto the electrosensory lateral line lobe, which in turn projects onto the torus semicircularis in the midbrain (Figure 1B). Midbrain afferents include both depressing and nondepressing synapses that converge onto individual midbrain neurons. We use three categories of stimuli global stimuli (social signals that stimulate the entire sensory surface simultaneously), a localized moving bar, and a larger moving sinewave grating.

Our computational model explains how direction selectivity arises from known features of midbrain neurons of the weakly electric fish *Eigenmannia*. Our model reproduces and explains the surprising experimental result that global synchronous electrosensory oscillations experienced prior to a local moving stimulus enhance direction selectivity of the local stimulus in weakly electric fish [17]. Moreover the model captures the diversity of the directionally selective neurons in the midbrain with respect to the observed correlation between direction selectivity and a measure of short-term synaptic depression.

Our model has been adapted, and substantially simplified, from a previously published model of direction selectivity [22]. The Chance et al. model incorporates a large number of dynamic variables and fixed parameters, many of which, we show, are not needed to reproduce the phenomena. We have significantly simplified the model, capturing the relevant features with a minimal number of dynamic variables (two), and substantially fewer fixed parameters (16), making the model amenable for the purpose of system identification [38]. We have provided our model code, written in MATLAB, in Protocol S1.

We model the response of a neuron to a spatiotemporal stimulus as a cascade of four elements: the afferents, the synapses, the synaptic conductance, and the cell membrane. In our simplest formulation of the model, only the depressing synapses have state, i.e., dynamics that depend on *state variables* that “remember” the stimulus history. State variables “remember” the stimulus history in the sense that differential equations integrating the element's input determine how they evolve. The other elements in the cascade are all memoryless, i.e., do not depend on any history-dependent quantities. For such elements, the input at every moment completely determines their output at that moment.

Our minimal instantiation utilizes a single depressing synapse (which has a memory in that it exhibits activity-dependent reduction in efficacy) and a single nondepressing synapse (memoryless). Based on neurophysiological observations, the short-term synaptic depression dynamics involve two time constants, corresponding to faster and slower depression processes, each involving a single state variable. Chance et al. [22] attribute direction selectivity to the faster process of depression and propose that contrast adaptation might involve the slower process of depression. As evidence, they note that both contrast adaptation and the slower process of depression exhibit similar time scales. We use the more general term *sparsification*, in lieu of *contrast adaptation*, because the slow process is driven by any persistent stimulus that causes depression, not just to changes in contrast of the scene. We will build on Chance's observations by elucidating another role for activation of the slower process: enhancement of direction selectivity.

### 

#### The input stimulus.

The input to the system is the stimulus intensity as a function of time *t* and position *x*: *I*(*t*,*x*). We consider only four classes of stimuli or temporal sequences thereof: constant functions for all *x* and *t*; global synchronous oscillations in which *I*(*t*,*x*) varies sinusoidally with time, independent of *x*; moving sine gratings, used by Chance et al. to test their own model; and a moving sinusoidal pulse (with a 1.5 cycle period), analogous to a moving bar. Following Chance et al. [22] and others we normalize the intensity function so that *I*(*t*,*x*) ∈ [−1,1] with *I*(*t*,*x*) = 0 representing 50% grey.

The temporal sequence we use for a moving pulse stimulus is defined (for *t*_0_ ≤ *t* ≤ *t_f_*) as follows:

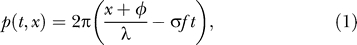


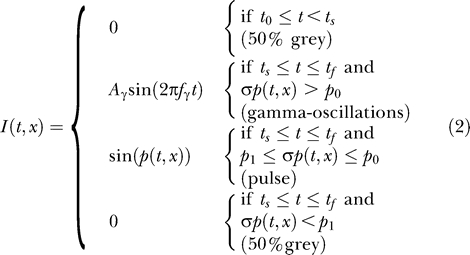



In Equation 1, λ is the spatial wavelength that sets the unit for space: without loss of generality, we say λ = 360°. Likewise *f* is the temporal frequency that determines the speed of the motion of the pulse. In this case, *f* determines more than just the scaling of time, because *f* interacts with the time constants of the model. The parameter *ϕ* determines the phase of the pulse in units of λ, in our case degrees. Finally σ = ±1 determines the direction of motion of the stimulus.

In Equation 2, the stimulus parameters *p*_0_ and *p*_1_ determine the moving pulse boundaries. Setting *p*_0_ − *p*_1_ = 3π gives the pulse a period of 1.5 cycles. The sign σ appears in the conditions on the right-hand side of Equation 2 to reverse the direction of inequalities, needed because *p* decreases with time for a positively directed stimulus (σ = 1) and increases with time for a stimulus in the opposite direction (σ = −1). We choose *p*_0_ and *p*_1_ so that sin(*p*_0_) = sin(*p*_1_) = 0, cos(*p*_0_) = σ, and cos(*p*_1_) = −σ to determine a time-symmetric pulse that darkens from 50% grey on its boundaries.

The parameters *t*_0_ and *t_f_* are, respectively, the initial and final times of the stimulus. The stimulus parameter *t_s_* is a switching time between stimuli in the temporal sequence. At time *t_s_* the moving pulse appears, usually outside, but moving toward, the model cell's receptive field. Ahead of the pulse (and also appearing at time *t_s_*) lies initializing input. Behind the pulse lies 50% grey. The initializing input consists of either global synchronous oscillations in the gamma band (*A*_γ_ > 0, 20 Hz ≤ *f*_γ_ ≤ 80 Hz) or 50% grey (*A*_γ_ = 0).

To compare our simulated responses with experimental data we include a preinitialization phase with 50% grey (*t*_0_ ≤ *t* < *t_s_*), prior to the gamma oscillations. The preinitialization phase allows a meaningful quantification (independent of the model's arbitrary initial state) of short-term synaptic depression. This quantification compares the model's response to the first cycle of global synchronous oscillations following preinitialization to the response to a later cycle after the gamma oscillations have affected the state of the model.

Equations 1 and 2 can, for the appropriate choice of stimulus parameters, also define all the other stimuli we employ (see figure captions). For example, the persistent sine grating (with a initialization before first cycle) can be defined by setting *p*_0_ = ∞ and *p*_1_ = −∞. Finally, note that we do not count the stimulus parameters as part of the 16 fixed parameters of the model neuron, because they determine properties of the stimulus rather than the model cell.

#### The afferents.

The afferents, the first element in our cascade, transform the input signal, *I*(*t*,*x*), into the firing rates, *R_d_*(*t*) and *R_n_*(*t*), of two pre-synaptic cells in the depressing and nondepressing channels, respectively. The receptive fields of these two cells are spatially separated, with centers at *x_d_* and *x_n_*. Following Chance et al. [22] receptive fields are separated by 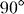 relative to the stimulus wavelength λ to maximize the model's response, however, the model is robust to the receptive field spacing relative to stimulus wavelength.

In the interest of model parsimony, we have replaced the kernel of the spatiotemporal integration from Chance et al. [22] with a delta function, rendering the afferents memoryless and confining the receptive fields to a single point. The input/output transformation of the afferents is given (for *I* ∈ (*d*,*n*}) by the rectified linear equation

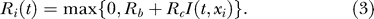



The parameters *R_b_* and *R_c_* are, respectively, the baseline firing rate and the contrast-dependent rate factor.

#### The synapses.

These afferent firing rates (*R_d_*(*t*), *R_n_*(*t*)) serve as input to the second element, the synapses, consisting of parallel depressing and nondepressing channels. In the case of the nondepressing synapse, inputs are trivially passed to the output: *R_n_*(*t*) ↦ *R_n_*(*t*). The depressing synapse, on the other hand, is the only component of our model with state. Its input/output relationship is given by the transformation *R_d_*(*t*) ↦ (*R_d_*(*t*),*D*(*t*),*S*(*t*)) where (*D*(*t*),*S*(*t*)) is the synapse state which evolves according to

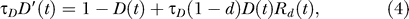


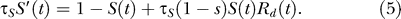



Here *τ_D_* and *τ_S_* are the fast and slow time constants of depression and *d* and *s* are the fast and slow depression strengths, taken to be between 0 and 1. In Chance's model, *D*(*t*) and *S*(*t*) are reduced by the factors *d* and *s*, respectively, subsequent to an action potential arriving at the corresponding depressing synapse, as timed by a Poisson process with inhomogeneous rate *R_d_*(*t*). Equations 4 and 5 result from averaging (taking the expected values of) the resulting stochastic differential equations, eliminating the randomness in the model. In our model, each depressing synapse is governed by two uncoupled, deterministic, nonlinear differential equations. Setting the depression strength *s* (or *d*) to 1 removes the corresponding state (after some input-independent transient); setting both strengths to 1 renders the synapse memoryless and equivalent to the nondepressing synapse.

#### The synaptic conductance.

The third element of the cascade is the model of the synaptic membrane conductance:

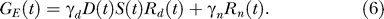



In the corresponding element of the Chance model, *G_E_*(*t*) is a state variable whose evolution is given by a differential equation with a time constant *τ_E_* = 2 msec, two orders of magnitude faster than the time constants *τ_D_* and *τ_S_* of our model. In Equation 6 we replace Chance's dynamic state variable with its asymptotic value, removing the memory of the element. This manipulation has only a small effect on the overall dynamics, a difference that is not important for reproducing the phenomena considered here.

We call the coefficients *γ_d_* and *γ_n_* the *synaptic factors*, depressing and nondepressing respectively. The term “synaptic factors”, in place of the more common “synaptic weights”, suggests a second more biologically relevant interpretation of the model. In this interpretation, each synapse in fact represents a synapse *class*, encompassing the contribution of a population of similar synapses that project onto the same midbrain neuron. The synapses within each class are identical in the sense that they have identical properties, receive identical input, and maintain an identical state. Under this interpretation, the synaptic factor equals the product of the individual synaptic weights and the number of synapses in the respective class. By covarying these two new parameters, class population and synaptic factor, the model demonstrates the diversity in the population of toral neurons that was observed by Ramcharitar et al. [17].

#### The membrane: Membrane and action potentials.

We consider three alternatives for modeling the cell membrane. The first candidate is the classical leaky-integrate-and-fire mechanism where *V*_0_ is the resting potential, *V_E_* is the synaptic reversal potential, and *τ_m_* is the membrane time constant:

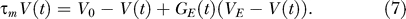



Equation 7 applies to the intervals between action potentials. We say the model fires an action potential when the membrane potential reaches a threshold potential *V_t_*. When an action potential occurs, the membrane potential discontinuously resets to *V_r_*, then again evolves according to Equation 7. By our choice of parameters *V*_0_ < *V_r_ < V_t_ < V_E_*, the synaptic current is excitatory.

Notice that the above formulation requires the membrane potential to be a state of the system. However, the time constant of the membrane is roughly an order of magnitude less than the time constant for the faster depression process, so we may again approximate this third state algebraically. This alternative to the leaky-integrate-and-fire mechanism replaces the membrane potential with its instantaneous asymptotic value given the present state of the conductance. This calculation averages the reversal potentials for the leak current (i.e., the resting potential) and the synaptic current:

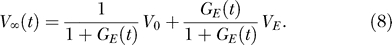



This approximation eliminates the reset dynamics to avoid chattering between *V_∞_*(*t*) and *V_r_* when *V_∞_*(*t*) > *V_t_*, limiting its use to modeling membrane potential with action potentials blocked.

Whereas the second alternative eliminates action potentials from the model, the third alternative predicts the action potential firing rate during these intervals. This firing rate can be fed to an inhomogeneous Poisson process to predict the timing of the action potentials within the periods of activity, or, more importantly, to determine a statistical model of the timing, useful for identifying the system from extracellular data [35,36]. Alternatives two and three can be combined in a model without adding an additional state variable to the system.

We say that the instantaneous firing rate of the neuron, assuming *V_∞_*(*t*) > *V_t_*, equals the reciprocal of the time required for the membrane potential to reach the action potential threshold, *V_t_*, from the reset potential, *V_r_*, assuming that the value of the synaptic conductance remains fixed at its present value, *G_E_*(*t*). If *V_∞_*(*t*) < *V_t_*, such a traversal cannot happen and we say the firing rate is 0. Because the membrane dynamics, given by Equation 7, are linear, the firing rate, as we have defined it, is an exponential function that can be calculated in closed form. Nevertheless, we find it helpful to make one further simplifying approximation. We assume that the rate of change of the membrane potential during the interval between reset and firing is approximately constant and equal to its value at threshold *V_t_*. The closer *V_r_* lies to *V_t_*, the better this approximation. With this simplifying assumption, our firing rate calculation reduces to a rectified linear algebraic equation:

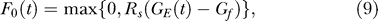
where

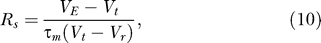


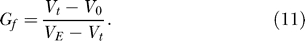



Here *R_s_* and *G_f_* are constants.

The quantity *F*_0_(*t*) represents the firing rate of the cell that is unbounded by a refractory period. Biological neurons have such bounds. For example, a refractory period of *τ_R_* puts an upper limit of 1/*τ_R_* on the firing rate of the cell. Without such a limit the firing rate of the model in response to certain stimuli can grow much larger than is biologically plausible. Fortunately by assuming the following saturation in firing rate, we can incorporate a refractory period into our model cell, without adding state to the membrane:

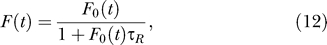
where *F*(*t*) is the firing rate output of the cell, bounded by the refractory period *τ_R_*. Note that if the firing rate *F*(*t*) determines the timing of action potentials by a Poisson process then it remains possible, though not particularly likely, that the cell will fire two (or more) action potentials within any given time interval of duration of *τ_R_*. This unlikely possibility will not prevent identifying the system through fitting parameters of the model to experimental data.

#### Measures of the response.

Finally, we consider several measures to quantify the response of the model to various stimuli. For the sine wave grating we use the direction index as a function of stimulus cycle number. Our calculation of direction index involves the expected number of action potentials for the *j*th cycle in the preferred (*P_j_*) and non-preferred (*N_j_*) directions, calculated by integrating the firing rate over the corresponding period. If we assume 0 ≤ *N_j_* ≤ *P_j_* and *P_j_* ≠ 0 (i.e., that the preferred direction has been correctly identified) then the following equation gives the direction index:

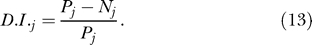



Note that the quantities *P_j_* and *N_j_* can be defined in other ways, but as long as 0 ≤ *Nj* ≤ *Pj* and *Pj* ≠ 0, the direction index lies between 0 and 1, reaches its maximum when *N_j_* = 0, and its minimum when *N_j_* = *P_j_*.

While the direction index remains the most common way to quantify direction selectivity, other quantifications can be useful. Ramcharitar et al. [17] used an unbounded measure of direction selectivity (referred to as *magnitude of direction selectivity*) to demonstrate a nearly linear correlation with a similar measure of short-term synaptic depression (called *magnitude of PSP depression*). To compare our results with these data, and because their experimental paradigm corresponds to the moving pulse stimulus for our model, we use this second measure to quantify the response of our model to the pulse stimulus. This second measure is the ratio (converted to dB) of the height of the depolarization of the membrane above the resting potential in the preferred direction to the same height in the non-preferred direction. A similar calculation quantifies short-term synaptic depression with the ratio of the response to the first cycle of global synchronous oscillations to a later cycle after the transient has decayed (PSP depression).

## Supporting Information

Protocol S1 MATLAB Code (.tar.gz file) MATLAB code with user-friendly interface for running Carver et al. model and plotting Figure 2.
(9 KB GZ).
